# Cochlear Implantation for Isoleucyl-tRNA Synthetase Mutation-Associated Mitochondrial Disease: A Case Report

**DOI:** 10.7759/cureus.67760

**Published:** 2024-08-25

**Authors:** Masaomi Motegi, Yuika Sakurai, Yasushi Mio, Toya Ohashi

**Affiliations:** 1 Department of Otolaryngology-Head and Neck Surgery, Gunma University Graduate School of Medicine, Maebashi, JPN; 2 Department of Otorhinolaryngology, The Jikei University School of Medicine, Minato, JPN; 3 Department of Anesthesiology, The Jikei University School of Medicine, Minato, JPN; 4 Department of Human Health Science and Therapeutics, The Jikei University School of Nursing, Chofu, JPN

**Keywords:** iras mutation, anesthesia complication, general anesthesia, diabetes mellitus, intellectual disability, profound sensorineural hearing loss, cochlear implantation, mitochondrial disease

## Abstract

Biallelic missense mutations in the nuclear-encoded, cytosolic isoleucyl-tRNA synthetase (IARS) gene are associated with a rare and complex multisystemic phenotype, including growth retardation, intellectual disability, muscular hypotonia, diabetes mellitus, and deafness. These mutations impact the cytosolic isoform of IARS, which plays a crucial role in protein synthesis. The pathogenesis involves mitochondrial dysfunction, despite IARS being primarily a cytosolic enzyme, potentially linking it to the observed clinical manifestations. The efficacy of cochlear implantation for deafness due to IARS mutations and the safety of general anesthesia in such patients remain unclear. This report describes a rare case of progressive sensorineural hearing loss caused by IARS mutation-associated mitochondrial disease, which was successfully treated with cochlear implantation. Additionally, we discuss the safety of general anesthesia in this patient. A Japanese woman with IARS mutation-associated mitochondrial disease was diagnosed with severe bilateral sensorineural hearing loss at five years of age and immediately received hearing aids. Her hearing progressively deteriorated to profound impairment, necessitating cochlear implantation at 26 years of age, which resulted in satisfactory hearing. Furthermore, no perioperative general anesthesia-related adverse events were reported. Our case demonstrates that cochlear implantation can effectively restore hearing. This suggests that sensorineural hearing loss caused by IARS deficiency is primarily due to cochlear dysfunction. This case demonstrated that hearing loss is a crucial feature of IARS mutation-associated mitochondrial disease, which can be mitigated by cochlear implantation. While general anesthesia can be safely administered, careful consideration of anesthetic choices, such as avoiding depolarizing muscle relaxants and prolonged use of propofol, is essential to prevent complications. In this case, general anesthesia was well tolerated without perioperative events, providing valuable insight into the potential safety of such procedures in similar patients. Nevertheless, further studies are needed to confirm these findings across a broader population.

## Introduction

Mitochondrial diseases result from impairments in the mitochondrial respiratory chain and are often characterized by hearing loss, which affects more than half of all patients throughout the course of their disease [1]. The final common pathway for this hearing impairment may involve adenosine triphosphate (ATP) deficits secondary to biochemical defects in the respiratory chain. The clinical characteristics of mitochondrial hearing loss vary widely. Although it is generally progressive with onset in childhood, many individuals are affected later in life [2]. In some cases, deafness is part of a multisystem disorder involving the central nervous, neuromuscular, or endocrine systems, while in other cases, it may present as an oligosyndromic disease [3].

To date, only three cases of cytosolic leucyl-tRNA synthetase (IARS) mutations have been documented globally [4]. Among these is our own case, involving a female patient with mitochondrial disease and hearing impairment, whose IARS mutation was identified through exome sequencing. For deafness in patients with mitochondrial disease, cochlear implantation is primarily considered when hearing aids are ineffective [5,6]. However, the efficacy of cochlear implantation in cases of IARS mutation-associated mitochondrial disease remains unclear. Anesthesia management in patients with mitochondrial diseases poses significant challenges due to their potential metabolic complications and sensitivities to specific anesthetic agents. It is crucial to understand these risks to ensure safe perioperative care. This is particularly important in complex cases where multiple organ systems are affected, requiring careful coordination and individualized treatment strategies.

This study presents an exceptionally rare case of progressive sensorineural hearing loss attributed to a mitochondrial disease associated with an IARS mutation, which was effectively managed through cochlear implantation. Additionally, we discuss the safety of general anesthesia in this patient, with reference to published considerations on anesthetic techniques that are deemed safer for patients with mitochondrial disorders.

## Case presentation

A Japanese woman born at 38 weeks of gestation presented with a complex multisystem phenotype, including prenatal-onset growth retardation, intellectual disability (verbal and performance IQs of 47 and 50, respectively, at 21 years), hepatopathy with fibrosis and steatosis, diabetes mellitus, epilepsy, and zinc deficiency. At five years of age, she was diagnosed with severe sensorineural hearing loss and was fitted with bilateral hearing aids. Decreased complex I activity was observed in her fibroblasts. Exome sequencing revealed biallelic IARS variants (NM_002161.5: c. 760C>T; p. Arg254*/c. 1310C>T, p. Pro437Leu) [4].

**Figure 1:**
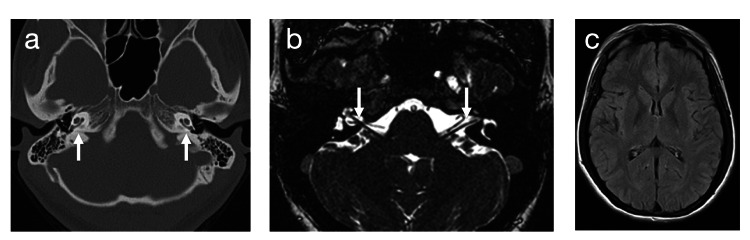
Computed tomography (CT) and magnetic resonance imaging (MRI). (a) Axial CT showing no abnormal morphology in both inner ears. Arrows indicate the cochleae on both sides. (b) Axial MRI showing intact bilateral cochlear nerves using fast imaging employing a steady-state acquisition technique. Arrows indicate the cochlear nerves within the internal auditory canals on both sides. (c) Axial T1-weighted MRI showing no evidence of encephalopathy.

**Figure 2:**
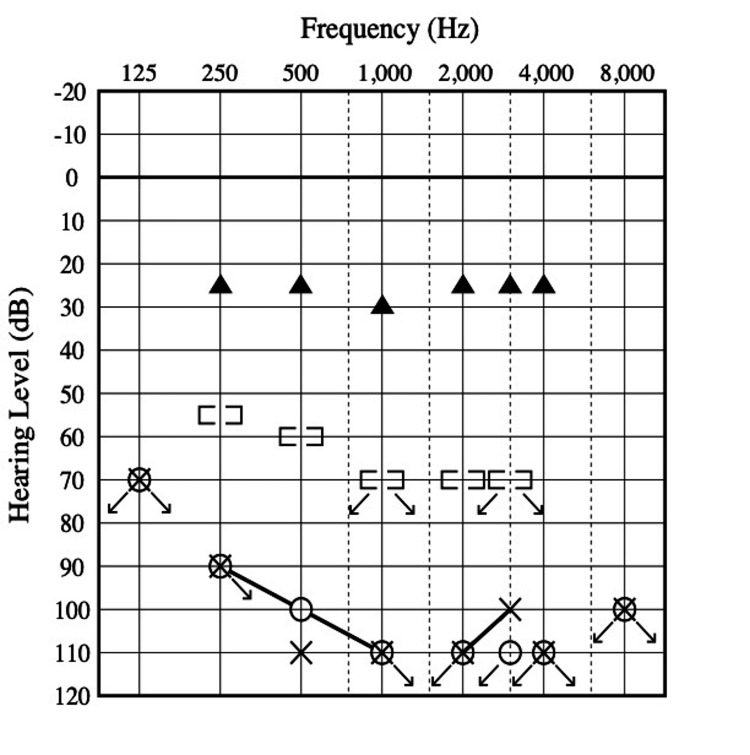
Audiograms before and after cochlear implantation. Pure-tone audiogram before cochlear implantation showing bilateral profound sensorineural hearing loss, with an average of 108 dB (without hearing aids). (○) right ear air-conduction hearing threshold; (×) left ear air-conduction hearing threshold; ([)right ear bone-conduction hearing threshold; (]) left ear bone-conduction hearing threshold. Right ear sound-field threshold after cochlear implantation shows significant improvement with hearing thresholds of 25 dB (▲ = sound source 1 m away).

Her hearing loss gradually worsened, and by 25 years of age, hearing aids had become ineffective, necessitating written communication or speech-reading. She was admitted to our Department of Otorhinolaryngology for cochlear implantation. Computed tomography (CT) and magnetic resonance imaging (MRI) revealed no abnormalities in the inner ear, cochlear nerve, or brain (Figure 1). Audiometry indicated bilateral profound hearing loss (108 dB) (Figure 2). Distortion-product otoacoustic emissions were poorly developed. Speech recognition, measured using a Japanese word list (67S word list), yielded a score of 0% for closed-set listening in the monosyllable perception test, even with hearing aids.

At 26 years of age, she underwent cochlear implantation (Nucleus 7® CI622; Cochlear LTD, Lane Cove, Australia) in the right ear. General anesthesia was induced with 100 mg fentanyl, 2 mg midazolam, and propofol. Propofol was administered via target-controlled infusion. She was intubated after an injection of 30 mg of rocuronium. Anesthesia was maintained with 0.15-0.2 mg/kg/min of remifentanil and propofol, with a propofol effect-site concentration of 3.2-4 mg/mL to maintain a bispectral index of 40-60. Sugammadex was used for rocuronium reversal. No perioperative anesthesia-related adverse events were observed, including metabolic depression or propofol infusion syndrome. All cochlear implant electrodes were successfully inserted intracochlearly and were functional after activation of the device. The sound-field threshold indicated a good response two months postoperatively (Figure 2). Three years after implantation, the patient’s speech recognition score in the monosyllable perception test was 65%. Two years postoperatively, she exhibited satisfactory improvement in communication, although she occasionally required speech-reading.

This study was approved by the Institutional Review Board affiliated with The Jikei University School of Medicine. Informed consent was obtained from the patient. This study was conducted according to the principles of the Declaration of Helsinki and its later amendments.

## Discussion

This study demonstrates the successful treatment of progressive sensorineural hearing loss in a patient with IARS mutation-associated mitochondrial disease through cochlear implantation, a novel approach not previously documented in the literature. Our findings are significant, as they suggest that cochlear dysfunction may be a primary contributor to hearing loss in such cases and that cochlear implantation has the potential to effectively restore auditory function.

This case provides valuable insights into the management of hearing loss in mitochondrial disorders, highlighting the potential of advanced genetic diagnostics and targeted surgical interventions to improve patient outcomes. tRNA synthetase deficiency represents a growing category of genetic diseases characterized by tissue-specific, predominantly neurological phenotypes. Mutations in cytosolic IARS have been implicated in several human diseases. To date, only three patients with biallelic missense mutations in IARS have been described worldwide [4]. These patients presented with a complex multisystemic phenotype, including prenatal-onset growth retardation, intellectual disability, muscular hypotonia, hepatopathy with fibrosis and steatosis, diabetes mellitus, zinc deficiency, and deafness. However, the pathogenesis underlying auditory dysfunction and optimal treatment strategies remain unclear.

Sensorineural hearing loss caused by mitochondrial dysfunction is predominantly attributable to cochlear dysfunction. In the cochlea, stria vascularis and hair cells are particularly vulnerable to respiratory chain defects and the resulting relative deficiency of intracellular adenosine triphosphate due to their high metabolic activity [7,8]. The spiral ganglion cells of the cochlear nerve are generally preserved in mitochondrial diseases [9,10]. Cochlear implants directly transmit sound signals to the cochlear nerve via electrical stimulation through the implant electrodes, effectively restoring hearing [5,6,11,12].

In some patients with multisystem mitochondrial disorders, the auditory system may be affected at the brainstem, midbrain, or cortical levels. However, we did not observe these impairments in our patient, suggesting that the cochlea plays a significant role in deafness associated with IARS deficiency. While previous reports have focused primarily on the genetic and phenotypic aspects of IARS mutations [4], our study adds a practical therapeutic perspective by demonstrating the viability of cochlear implantation as a treatment for hearing impairment in these patients.

High-concentration anesthetics used for general anesthesia can inhibit mitochondrial function in vitro [13]. Inhalation anesthetics inhibit complex I [14], and benzodiazepines may exert inhibitory effects on adenosine nucleotide translocase [15]. As a result, these agents could affect energy production and induce fatal metabolic depression in patients with mitochondrial disease. However, when used within recommended doses, they have not been associated with severe anesthesia-related morbidity or mortality in adults with mitochondrial diseases [16]. The safety of propofol in mitochondrial diseases is not well established [17], although its safe use in children with these conditions has been reported [18,19]. While prolonged propofol use is presumed to be the cause of propofol infusion syndrome [18], we used it for both induction and maintenance of anesthesia to avoid postoperative nausea and vomiting, which positively influences glycemic control in insulin-dependent diabetes. Nevertheless, the anticipated prolonged use of propofol may necessitate the use of volatile anesthetics instead [17,18].

The safety of various muscle relaxants in mitochondrial disease remains unclear; however, depolarizing muscle relaxants (e.g., succinylcholine) may induce malignant hyperthermia [20]. Therefore, non-depolarizing muscle relaxants (e.g., rocuronium) are recommended. Nonetheless, individual assessment is crucial to prevent hyperglycemia or lactic acidosis, given the diverse nature of mitochondrial diseases. Our case involving insulin-dependent diabetes underscores the importance of individualized anesthesia management in patients with mitochondrial disorders.

The absence of perioperative adverse events in our patient suggests that, with careful selection and monitoring of anesthetic agents, surgical interventions can be performed safely. This finding aligns with existing recommendations and contributes to the broader understanding of anesthesia safety in mitochondrial diseases [16,19].

This case report has several limitations. First, the rarity of IARS mutation-associated mitochondrial disease limits the generalizability of our findings. Additionally, further research is needed to evaluate the long-term efficacy and safety of cochlear implantation in such patients. Despite these limitations, our approach demonstrates significant strengths. The successful cochlear implantation in this case highlights a viable treatment option that can substantially improve auditory function and quality of life in patients with mitochondrial diseases.

Future research should focus on larger cohort studies to validate these findings and explore the long-term outcomes of cochlear implantation in patients with mitochondrial diseases. Further investigations into the underlying pathophysiology of IARS mutations and their impact on auditory function could provide deeper insights into optimizing treatment strategies for these rare disorders.

## Conclusions

This case report illustrates the effectiveness of cochlear implantation in restoring progressive sensorineural hearing loss in a patient with mitochondrial disease associated with mutations in the IARS gene, which has two isoforms - both of which can contribute to mitochondrial dysfunction. This intervention significantly improved the patient's auditory function and quality of life. Additionally, the safe administration of general anesthesia, with careful perioperative management, underscores the feasibility of surgical procedures in patients with this type of mitochondrial disorder. Given the complexity and rarity of IARS-associated mitochondrial disease, this case offers valuable insights into the clinical management of mitochondrial diseases and emphasizes the potential of cochlear implants to significantly enhance the quality of life for affected individuals.
